# Parotid gland radiation dose‐xerostomia relationships based on actual delivered dose for nasopharyngeal carcinoma

**DOI:** 10.1002/acm2.12327

**Published:** 2018-04-17

**Authors:** Jingjiao Lou, Pu Huang, Changsheng Ma, Yue Zheng, Jinhu Chen, Yueqiang Liang, Hongsheng Li, Yong Yin, Danhua Liu, Gang Yu, Dengwang Li

**Affiliations:** ^1^ Shandong Province Key Laboratory of Medical Physics and Image Processing Technology Institute of Biomedical Sciences School of Physics and Electronics Shandong Normal University No.88, Wenhua East Road Jinan 250014 China; ^2^ Shandong Cancer Hospital Affiliated to Shandong University Shandong Academy of Medical Science No.440, Jiyan Road Jinan 250117 China

**Keywords:** cone‐beam CT, deformable image registration, nasopharyngeal carcinoma, xerostomia

## Abstract

Xerostomia induced by radiotherapy is a common toxicity for head and neck carcinoma patients. In this study, the deformable image registration of planning computed tomography (CT) and weekly cone‐beam CT (CBCT) was used to override the Hounsfield unit value of CBCT, and the modified CBCT was introduced to estimate the radiation dose delivered during the course of treatment. Herein, the beams from each patient's treatment plan were applied to the modified CBCT to construct the weekly delivered dose. Then, weekly doses were summed together to obtain the accumulated dose. A total of 42 parotid glands (PGs) of 21 nasopharyngeal carcinoma patients were analyzed. Doses delivered to the parotid glands significantly increased compared with the planning doses. V_20_, V_30_, V_40_, D_mean_, and D_50_ increased by 11.3%, 28.6%, 44.4%, 9.5%, and 8.4% respectively. Of the 21 patients included in the study, eight developed xerostomia and the remaining 13 did not. Both planning and delivered PG D_mean_ for all patients exceeded tolerance (26 Gy). Among the 21 patients, the planning dose and delivered dose of D_mean_ were 30.6 Gy and 33.6 Gy, respectively, for patients with xerostomia, and 26.3 Gy and 28.0 Gy, respectively, for patients without xerostomia. The D_50_ of the planning and delivered dose for patients was below tolerance (30 Gy). The results demonstrated that the p‐value of V_20_, V_30_, D_50_, and D_mean_ difference of the delivery dose between patients with xerostomia and patients without xerostomia was less than 0.05. However, for the planning dose, the significant dosimetric difference between the two groups only existed in D_50_ and D_mean_. Xerostomia is closely related to V_20_, V_30_, D_50_, and D_mean_.

## 1 INTRODUCTION

Oral complications induced by radiotherapy are universal in the treatment of head and neck carcinomas (HNC). Xerostomia results from the deleterious effect of radiation, and is the most common persistent oral sequela for HNC patients who receive therapeutic doses.^1^ It occurs with difficulties in swallowing and speaking, loss of taste, and dental caries, which have a detrimental impact on the quality of life.^2^ The occurrence of xerostomia is related to several factors such as the radiation dose, the volume of irradiated tissue, and the use of concurrent radiation sensitizing and chemotherapy drugs. Much research has been performed on delivering radiation to normal tissue to spare patients from the development of complications. Intensity‐modulated radiation therapy (IMRT) has been considered as the standard therapeutic technique of HNC because of its highly conformal, modulated techniques.^3^, ^4^ It permits the delivery of a high dose to the target volume and spares the surrounding critical structures. Several studies have noted that IMRT has advantages over three‐dimensional conformal radiotherapy in terms of sparing the parotid gland (PG), and it can improve the quality of life for HNC patients.^5^, ^6^ However, position variations and anatomical changes over the course of treatment limit the benefits of IMRT and lead to a clinically significant dose difference between the initial plan and delivery.

Previous studies have shown that anatomy changes may cause more dose deviations in organs at risk (OARs) than in target volumes.^7^, ^8^, ^9^, ^10^ It is true that changes in anatomy were found among most of the nasopharyngeal carcinoma (NPC) patients.^11^ Generally, bodyweight loss, primary tumor shrinkage, and PG volume reduction could induce a PG dose change and increase the risk of xerostomia. In order to correct these deviations, adaptive radiotherapy (ART), online re‐optimization, or offline replanning is recommended.^12^, ^13^ Castelli et al. investigated the impact of ART to spare the PG and decrease the risk of xerostomia using weekly computed tomography (CT), and included the PG overdose, the benefit of ART, and anatomical markers related to dose differences between the planning and accumulated dose (with/without replanning).^2^ Although the time and the optimal number of replanning incidents remain unclear and the strategies of replanning remain uncertain, the benefits of ART have previously been demonstrated.^14^ However, it is not likely that patients can obtain CBCT scans for every fraction during the entire treatment, and not every patient will benefit from ART. Thus, identifying patients earlier by selection criteria to spare OARs and further decrease the risk of complication will be beneficial to both patients and radiation departments. Previous studies selected patients by determining some criteria that would allow them to benefit from ART or replanning.^4^, ^15^, ^16^

In clinical practice, CT is the only imaging modality that can be used for treatment planning. Cone‐beam CT (CBCT) integrated into linear accelerators cannot be used as a planning imaging modality because of its inferior image quality, poor electron density accuracy, scatter, and motion artifacts. However, CBCT scans possess the position and shape information of the target and organs at risk in real time. Daily or weekly on‐board CBCT is used to assist patient setup. In addition, CBCT data can also potentially be used for dose reconstruction with the electron density calibrated. Yang et al. and Ding et al. studied the modified CBCT (mCBCT)‐based dose reconstruction method and validated that it allowed for acceptable dosimetric evaluation.^17^, ^18^ Furthermore, Hunter et al. already determined the PG dose‐effect using weekly CBCT.^14^

In our previous study, we investigated dosimetric variations of the liver using deformable registration of planning CT (pCT) and CBCT and found that there was an increase in D_50_ and D_mean_ when compared with the planning dose.^19^ Additionally, a significant dosimetric difference between patients with and without radiation‐induced liver disease (RILD) has been reported. Charlotte et al. stated in their review that only a few articles reported the clinical relevance of dosimetric changes in terms of complications of head and neck cancer patients.^16^ As for dosimetric variation, the delivered dose may not correspond to the planned dose. Several studies investigated PG dose variation and found great dosimetric changes.^10^, ^12^, ^20^ However, inaccuracies in the calculation of dosimetric change should not be ignored.

One of the reasons leading to the inaccuracies is that in most studies, treatment plans used to calculate dose distributions were constructed on CT acquired a few days before radiotherapy, such as repeated planning CT. Patients experience anatomical changes during the treatment course, and consequently, a plan based on pretreatment CT images cannot precisely reflect the actual dose distribution during fractionated IMRT. In that case, the correlation between clinical outcomes and dosimetric changes might not be accurate. Hence, a real‐time imaging modality such as CBCT is a better choice for estimating the delivered dose and investigating the relevance of dosimetric changes and complications.

Xerostomia might not occur in salivary gland tumors because the administered radiation is usually restricted to the ipsilateral gland. However, for the nasopharynx, the parotid glands are usually affected by radiation, and severe and permanent xerostomia may result. In this study, 21 NPC patients were included to investigate the association between dosimetric factors and xerostomia. We hypothesize that the actual delivered dose based on CBCT correlates more closely with the development of xerostomia than the planning dose, and some dosimetric characteristics between patients with xerostomia and patients without xerostomia should be different. This study aims to validate the hypothesis by using the mCBCT approach.

## 2 MATERIALS AND METHODS

### 2.A Patients and tumors

This retrospective study involved 21 NPC patients. The patients’ selection criteria were as follows: (1) patients enrolled in this study were treated with IMRT at the Radiation Department of the Shandong Cancer Hospital and Institute between August 2010 and August 2015; (2) a positive biopsy for nasopharyngeal carcinoma was obtained for all of the patients, and (3) there were no recurrent patients, nor had any undergone resection. Patients diagnosed with other malignances or treated with non‐IMRT techniques were excluded. Among the 21 patients, eight developed xerostomia but the other 13 did not. All parotid glands of patients taken into consideration were outside the PTVs. Patient and tumor characteristics of the initial plan are shown in Table 1. The study was approved by the Shandong Cancer Hospital ethics committee. All patients provided written informed consent.

**Table 1:** Characteristics of patients with xerostomia and patients without xerostomia

Characteristics	Patients with xerostomia (*n* = 8)	Patients without xerostomia (*n* = 13)
Gender		
Female	2	3
Male	6	10
Age (yr)		
Range	38–68	16–51
aKPS		
80	4	2
90	4	9
100	0	2
TNM stage		
T_1_N_2_M_0_	1	1
T_2_N_1_M_0_	0	2
T_2_N_0_M_X_	0	1
T_2_N_1_M_X_	0	1
T_2_N_2_M_0_	5	4
T_2_N_2_M_1_	0	1
T_3_N_2_M_0_	2	0
T_4_N_1_M_0_	0	1
T_4_N_2_M_0_	0	2
bPG volume (cc)		
Range	15.3–34.0	8.9–28.9

a KPS = Karnofsky performance status;

b PG = parotid gland.

### 2.B Treatment and planning

All patients underwent simulation on Philips CT Big Bore, immobilized in a supine position with a thermoplastic mask covering the head, neck, and shoulders. Intravenous contrast‐enhanced CT using 3 mm slice thickness was carried out, ranging from the vertex to the manubria sternal joint for planning. These data were transferred to the treatment planning system (Pinnacle, version 9.2 to 9.8). Targets were delineated by clinicians with the assistance of a combination of CT and MRI. Magnetic resonance scans were obtained using the same position of simulation. The delineation of organs at risk was completed by physicians. All patients’ radiation doses were planned and delivered using inverse IMRT. The prescribed radiation dose was 70 Gy at 2 Gy per fraction delivered over 6–7 weeks (5 or 6 fractions a week). The objective parameter used in IMRT optimization for the PG was at least one PG D_mean_ < 26 Gy or D_50_ < 30 Gy. All patients underwent CBCT scans with linear accelerators (Varian Trilogy) once a week to correct setup errors before radiotherapy. CBCT scanning was performed with 2.5 mm slice thickness. These CBCT scans were noted as CBCT week 1, week 2, week 3, and so on. Patients included in the study received chemotherapy with cisplatin and/or tegafur.

### 2.C Dose construction

A plan based on mCBCT was generated to calculate the delivered dose. First, weekly CBCT and pCT were registered using rigid registration, followed by deformable image registration (DIR) to obtain the correct Hounsfield unit (HU) value of CBCT. Then, beam configurations and dose constraints of the initial plan were reapplied to mCBCTs. The target volume and OAR delineations upon pCT were automatically propagated onto mCBCT using the deformation vector field resulting from the registration as described above. All automatically propagated delineations were checked by the same proficient physicist. Delineations would be revised if the mapping structures were not in accordance with the anatomy on the mCBCT unless the results satisfied the physicist. Last, the dose distribution was recalculated.

### 2.D Gradient‐based deformable image registration

3D/2D registration methods can be classified into three categories: extrinsic, intrinsic, or calibration‐based.^21^ The intrinsic methods can be further divided into two main categories, feature‐ and intensity‐based, and it is accepted that the latter has an important advantage over the former in that all available information can be utilized in the images. However, it is possible that registration would fail because of the inaccuracy of the CBCT intensity. Scatter is one of the main reasons for intensity inaccuracy. Many prior studies showed that the scattering of CBCT results in a low‐frequency signal. That is to say, in the scatter artifact images, the shapes of almost all inner and outer object boundaries, such as boundaries between bone, fat, muscle, and air, can be perceived.^22^ Based on the characteristics of scatter, we proposed the gradient‐based free‐form deformation algorithm (GFFD).^23^

The GFFD algorithm measures the similarity by using 3D gradient vector fields. The local polynomial approximation‐intersection of confidence intervals algorithm is performed to accommodate the image sampling anisotropy. A “bi‐directional” force along with an adaptive force strength adjustment is introduced to accelerate the effect of the convergence process. These strategies are expected to decrease the effects of the inconsistent intensities.

### 2.E Dose calculation and accumulation

**Figure 1:**
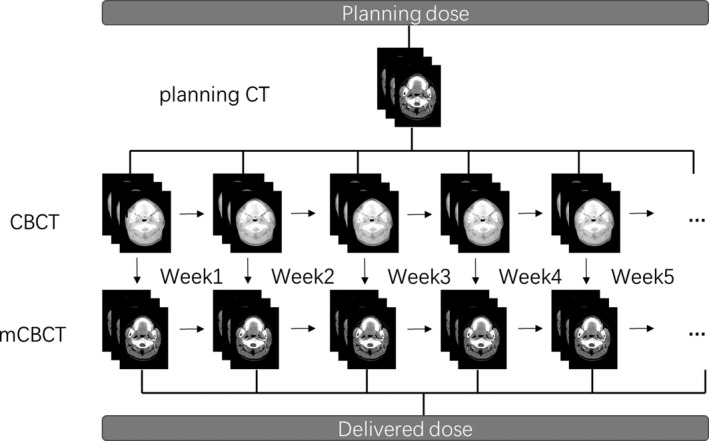
The derivation of planning dose and delivered dose.

**Figure 2:**
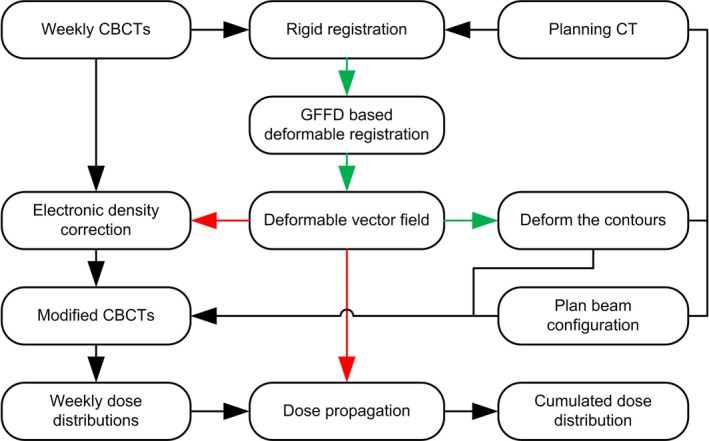
A flow chart detailing the forward and inverse registration steps in the study. The red arrows indicate the forward registration steps, and the green arrows indicate the inverse registration steps.

The planning dose was calculated at the time of treatment planning. The accumulated delivered dose was derived from the weekly CBCTs. Firstly, the weekly mCBCT was aligned and registered with the initial planning CT through rigid registration. Secondly, deformable registration was conducted to optimize the local regions. The corresponding registration fields, i.e., the inverse fields of GFFD, were used to propagate the daily doses. Each weekly mapped plan based on mCBCT was superimposed on the initial plan to estimate the cumulative delivered dose over the entire treatment course. The dose volume histogram was used to evaluate the V_20_, V_30,_ V_40_, D_50_, and D_mean_ of the PG. Figure 1 shows the derivation of the total planning dose and accumulated delivery dose. Figure 2 shows the forward and inverse registration steps.

### 2.F Statistical analysis

The paired samples *t*‐test was used to obtain the association between the planning dose and the actual delivered dose. The independent samples *t*‐test was used to determine whether there is a statistically significant dose difference between patients with xerostomia and patients without xerostomia. All analyses were performed using IBM SPSS version 23.0 software. A *P*‐value less than 0.05 was considered significant.

## 3 RESULTS

### 3.A Imaging and automatic propagation

**Figure 3:**
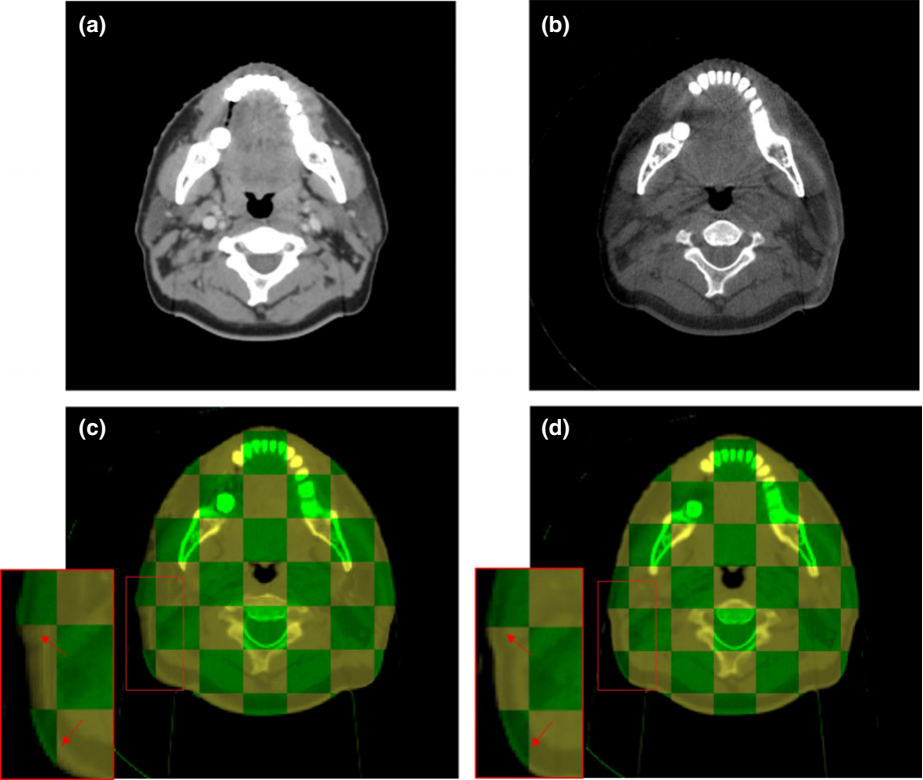
One example of the comparison between the GFFD algorithm and the Demons algorithm; (a) is the planning CT image; (b) is the corresponding CBCT image; (c) is the checkerboard comparison between the fixed image (CBCT) and the registered image (mCBCT) after Demons registration; (d) is the checkerboard comparison between the fixed image (CBCT) and the registered image (mCBCT) after GFFD registration. In (c) and (d), the checkerboard in green indicates the fixed image, and the checkerboard in yellow indicates the registered image.

Figure 3 shows the pCT and CBCT registration error of GFFD and Demons, respectively, for a head example. As can be seen from Figs. 3(c) and 3(d), the results of registration of GFFD are comparable to that of Demons, and they are even better than Demons in the regions marked by the red rectangle.

**Figure 4:**
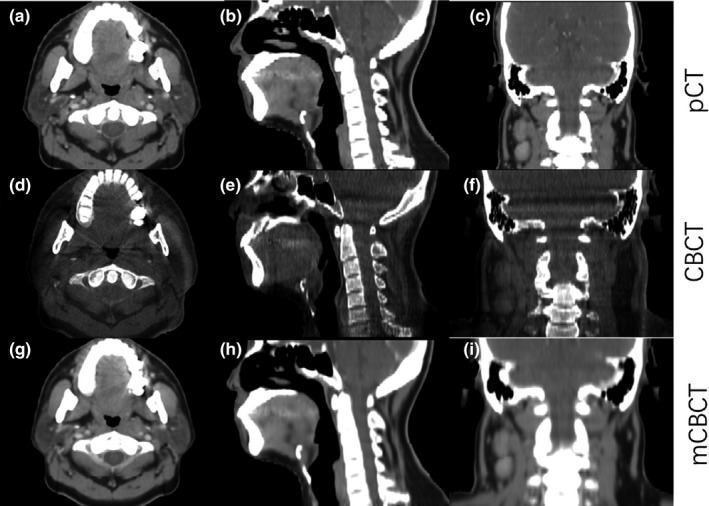
An example of the transverse plane, sagittal plane, and coronal plane of planning CT, CBCT, and mCBCT.

Figure 4 shows an example of the transverse plane, sagittal plane, and coronal plane, of planning CT, CBCT, and mCBCT respectively. As can be seen, the image quality of mCBCT contrasts well with CBCT, and it is comparable to that of pCT. There is less noise on the mCBCT as compared with CBCT. More importantly, the electron density of mCBCT is calibrated. Herein, accurate dose construction is allowed.

**Figure 5:**
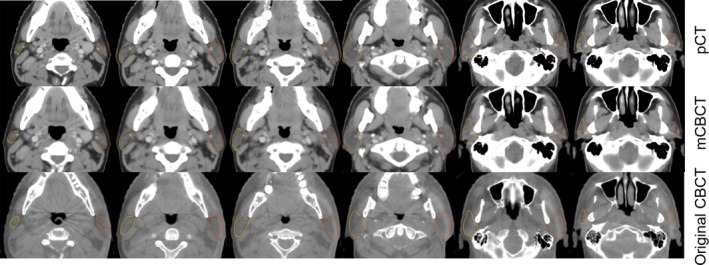
One example of automatically propagated parotid gland contours from planning CT to mCBCT. The last row shows the deformed contours overlaid on the original CBCT images. The yellow and brown line on CT and CBCT images represents the contour of the right and the left parotid gland respectively.

The inverse deformation field of registration mapped the contours on CBCT from pCT. Figure 5 illustrates the automatic propagation of the parotid gland of an NPC patient. The last row of Fig. 5 shows the deformed contours overlaid on the original CBCT images. As can be seen, the automatically generated contours on mCBCT match the structures on CBCT well, which allowed us to perform the delivered dose calculation.

### 3.B Comparison of planning and delivery dose

A total of 42 parotid glands of 21 NPC patients were analyzed. Among the total 42 PGs, 34 PGs experienced to an increase in cumulative delivered dose as compared with the planning dose, and eight PGs experienced a decrease. Nearly all patients had at least one gland that experienced an increase in the cumulative delivered dose compared to the planning dose. For the 42 PGs, the D_mean_ of the cumulative dose increased by 9.5% as compared with that of planning dose. D_50_, V_20_, V_30_, and V_40_ increased by 8.4%, 11.3%, 28.6%, and 44.4% respectively. The association between planning doses and actual delivered doses was determined with the paired samples *t*‐test (Table 2). From the results, we can see that the V_20_ values between planning doses and delivered doses are correlated with each other. The same situation applies to V_30_, V_40_, D_50_, and D_mean_. Additionally, the p‐values of the above dosimetric parameter differences between the planning doses and the delivered doses were all less than 0.05. The delivered doses dramatically increased compared with the planning doses.

**Table 2:** The differences between planning dose and delivered dose

Parameters	Planning	Delivery	Correlation	P value
^1^V_20_ (%)	67.5 ± 21.8	73.2 ± 21.6	0.919	<0.01
V_30_ (%)	31.7 ± 14.0	38.2 ± 15.2	0.747	<0.01
V_40_ (%)	18.0 ± 10.0	22.5 ± 10.9	0.657	<0.01
^2^D_50_ (Gy)	23.8 ± 5.2	25.9 ± 5.8	0.789	<0.01
D_mean_ (Gy)	27.9 ± 5.4	30.1 ± 5.9	0.809	<0.01

^1^V_20_ = the percentage of parotid gland volume that received 20 Gy in the total parotid gland.

^2^D_50_ = dose to the 50% of the parotid gland.

### 3.C Dosimetric parameters correlated with xerostomia

**Figure 6:**
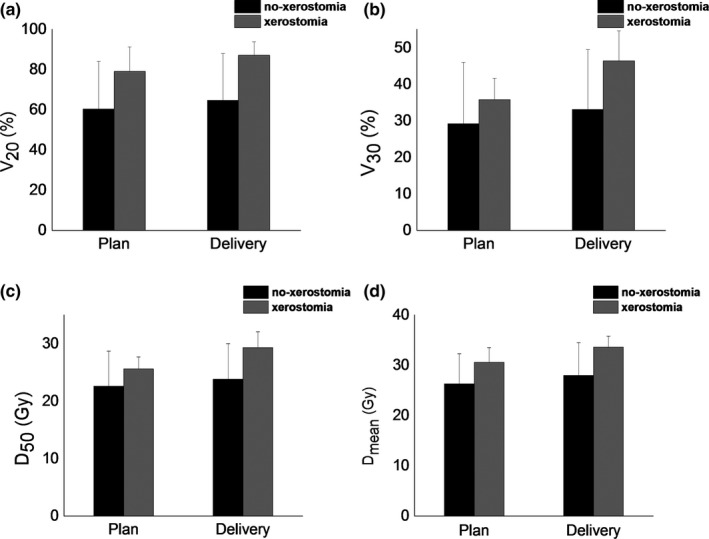
The reconstructed (a) V_20_, (b) V_30_, (c) D_50_, and (d) D_mean_, relative to the plan. Error bars indicate the standard deviation. Herein, no‐xerostomia represents patients without xerostomia, and xerostomia represents patients with xerostomia.

Of the 21 patients included in the study, eight developed xerostomia, while the rest of the patients did not. PG V_20_, V_30_, V_40_, D_50_, and D_mean_ between patients with xerostomia and patients without xerostomia were compared. The dosimetric parameters of planning doses and delivery doses for patients with xerostomia and patients without xerostomia are presented in Tables 3 and 4. The results indicate that the PG D_mean_ of planning doses for all patients exceeded the PG tolerance (26 Gy). The same situation applies to the PG D_mean_ of delivered doses. Among the 21 patients, the PG D_mean_ of planning doses and delivered doses were 30.6 Gy and 33.6 Gy, respectively, for patients with xerostomia, and 26.3 Gy and 28.0 Gy, respectively, for patients without xerostomia. PG D_mean_ over parotid gland tolerance (26 Gy) was observed in 16 patients (16/21). The D_50_ values of the planning doses and the delivered doses for all patients were below the PG tolerance (30 Gy). V_20_, V_30_, V_40_, D_50_, and D_mean_ of the planning doses for patients with xerostomia were all higher than those of patients without xerostomia. The same situation applied to V_20_, V_30_, V_40_, D_50_, and D_mean_ of the delivered doses. The p‐values of V_20_, V_30_, D_50_, and D_mean_ difference of the delivery dose between patients with xerostomia and patients without xerostomia are less than 0.05. However, for the planning doses, the significant dosimetric difference between the two groups is only embodied in D_50_ and D_mean_. Xerostomia is closely related to V_20_, V_30_, D_50_, and D_mean_. The details of V_20_, V_30_, D_50_, and D_mean_ for the two groups are shown in Fig. 6.

**Table 3:** Planning dose characteristics betweent patients with xerostomia and patients without xerostomia

Parameter	^1^Ref	Patients with xerostomia (*n* = 8)	Patients without xerostomia (*n* = 13)	*P* value
^2^V_20_ (%)	NA	79.1 ± 12.2	60.4 ± 23.6	0.06
V_30_ (%)	NA	35.8 ± 5.8	29.2 ± 16.8	0.08
V_40_ (%)	NA	20.3 ± 4.0	16.5 ± 12.2	0.16
^3^D_50_ (Gy)	30	25.6 ± 2.1	22.6 ± 6.1	0.03
D_mean_ (Gy)	26	30.6 ± 2.9	26.3 ± 6.0	<0.01

^1^Ref = parotid radiation tolerance.

^2^V_20_ = the percentage of parotid gland volume that received 20 Gy in the total parotid gland.

^3^D_50_ = dose to the 50% of the parotid gland.

**Table 4:** Delivery dose characteristics between patients with xerostomia and patients without xerostomia

Parameter	^1^Ref	Patients with xerostomia (*n* = 8)	Patients without xerostomia (*n* = 13)	*P* value
^2^V_20_ (%)	NA	87.1 ± 6.7	64.7 ± 23.3	<0.01
V_30_ (%)	NA	46.4 ± 8.2	33.1 ± 16.4	0.05
V_40_ (%)	NA	27.6 ± 6.9	19.3 ± 11.7	0.06
^3^D_50_ (Gy)	30	29.3 ± 2.8	23.8 ± 6.2	<0.01
D_mean_ (Gy)	26	33.6 ± 2.2	28.0 ± 6.5	<0.01

^1^Ref = parotid radiation tolerance.

^2^V_20_ = the percentage of parotid gland volume that received 20 Gy in the total parotid gland.

^3^D_50_ = dose to the 50% of the parotid gland.

## 4 DISCUSSION

The algorithm of DIR has an impact on the results of this study because the HU value of mCBCT overridden by DIR would influence the accuracy of dose construction. Additionally, the DIR algorithm determines the accuracy of propagation of structural contours. We have researched the deformable registration algorithm over the last few years.^24^, ^25^ We also researched automatic contouring based on segmentation for radiotherapy treatment planning.^26^, ^27^, ^28^ We found that intensity‐based deformable registration algorithms are susceptible to distorting tissues because the presence of inconsistent intensities between CT and CBCT could cause significant registration errors. The DIR algorithm performed in this study was presented in our previous work, and was tested using phantom and clinical data, which demonstrated the significance of the registration method.^23^ We assessed the volume overlapping using the Dice Similarly coefficient (DSC) in that study. The DSC value between the contours propagated by the GFFD algorithm and the contours edited by the oncologist was 96% for the parotid gland.

Many studies have been devoted to investigating the side effects of radiotherapy in head and neck cancer. This is mainly because OARs and tumor target volumes of HNC are close to each other.^16^ The parotid gland is the most studied organ at risk in head and neck cancer. Most of the previous studies reported on anatomic and dosimetric changes of the PG. This trend of study can be explained by the fact that radiation dosimetric changes for PGs are associated with saliva reduction and xerostomia.^29^, ^30^, ^31^

CT scans for radiotherapy planning are acquired prior to treatment. Generally, patient posture and anatomic change occur during the course of radiotherapy. Hence, the dose actually delivered to the patient often differs from that of the initial plan. Charlotte et al. reviewed the literature published in the last decade and pointed out that, on average, the PG mean dose increase was 2.2 ± 2.6 Gy as compared to the dose calculated on the planning CT.^16^ They also emphasized that the largest PG dose increase found by Chen et al. and Cheng et al. was 10.4 Gy in the sixth week of radiotherapy, and the largest median dose was 7.8 Gy at the 25th fraction.^10^, ^20^ These results are approximately consistent with ours. Our results showed that the average PG mean dose increase was 2.2 Gy ± 3.5 Gy, ranging from −6.7 to +8.6 Gy.

Radiologists usually concentrate more on the constraints of D_mean_ and D_50_ for the PG, but ignore V_20_, V_30_, and V_40_, which could possibly lead to the development of xerostomia. The dose delivered to patients is usually different from that of planning because of position error and anatomical variation. In this study, the dose delivered to patients dramatically increased as compared with the planning dose. Additionally, it was found that the V_20_, V_30_, D_50_, and D_mean_ of delivery doses for the PG between patients with xerostomia and patients without xerostomia were different. However, only D_50_ and D_mean_ for the PG were found to be different between the two groups while considering the planning doses. It is apparent that the difference of V_20_ and V_30_ between the two groups will not be detected if we only consider the dosimetric consequences based on initial plans. To some extent, it emphasizes the significance of CBCT‐based image‐guided radiotherapy for patient positioning and planning optimization.

A number of studies have described PG anatomic or dosimetric changes, but only a few studies have reported the clinical relevance of these factors with regard to complications. Several studies found that PG shrinkage could increase the incidence of complications, while Sanguineti et al. gave the opposite conclusion.^32^, ^33^, ^34^ Hunter et al. investigated the association of planning/delivery PG dose and salivary outcome, and he concluded that the associations of planning/delivery dose and salivary outcome were significant, but the relationship between dosimetric change and saliva flow was not strong.^14^ In the current study, we estimated the actual delivered dose by using weekly mCBCT and compared the dosimetric difference between patients with xerostomia and patients without xerostomia. Many studies reported factors that were correlated with dosimetric changes, such as weight loss, neck thickness, PG volume loss, and center of mass (COM) shift. It showed that PG volume loss significantly correlated with the dose deviation from the planning dose. Three large studies reported a significant correlation of PG dose with PG volume loss.^34^, ^35^, ^36^ However, on average, the relationship between these factors and dosimetric changes was still unclear.

Generally, PG volume will decrease after radiotherapy. Previous studies suggested that, on average, the PG volume reduction rate was 26 ± 11%.^16^ In our study, the PG volume reduction rate was 13.5% and 13.2%, respectively, for patients with xerostomia and patients without xerostomia (Table 5). Although there is little difference in the PG volume reduction rate between patients with xerostomia and patients without xerostomia, the dose deviations between the two groups greatly differ. More study is needed to explore this issue.

**Table 5:** The change of parotid gland volume for patients with xerostomia and patients without xerostomia

Time	Patients with xerostomia (*n* = 8)	Patients without xerostomia (*n* = 13)
Pretreatment PG volume (cc)	22.6 ± 6.9	14.6 ± 4.3
Post‐treatment PG volume (cc)	18.3 ± 4.1	11.8 ± 2.0
Volumetric change (%)	13.5 ± 23.5	13.2 ± 24.4

**Figure 7:**
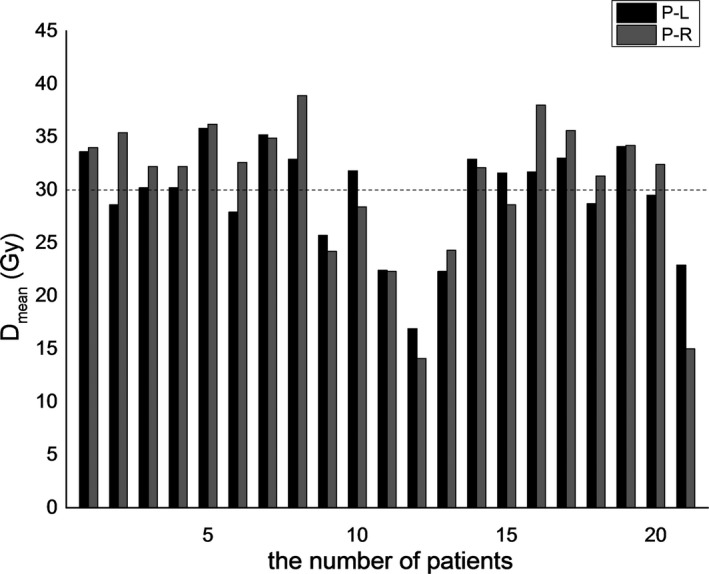
Delivered parotid gland D_mean_ for 21 patients. Herein, P‐L represents the left parotid gland, and R‐L represents the right parotid gland. No. 1 to No. 8 patients developed xerostomia, and the remaining 13 did not. The dotted line along 30 Gy represents the tolerance of the parotid gland.

It is worth mentioning that although the D_mean_ value of several patients without xerostomia was over the tolerance of 30 Gy, some were even larger than that of patients with xerostomia but they did not develop xerostomia (Fig. 7). Perhaps it is because xerostomia can be caused by several other factors such as physical status, age, and oral nursing care. In addition, we found that elder patients were at high risk to suffer xerostomia. The average age of patients with xerostomia and patients without xerostomia is 53.2 and 33.4 yr old (*P* = 0.02) respectively.

Radiotherapy‐induced xerostomia is the result of the deleterious effect of radiation, and it is necessary to monitor the dose delivered to the PGs. To some extent, severe and acute xerostomia can be combated and even be prevented if the dose to the PGs is limited or even decreased. On one hand, radiation protocols designed for the parotid gland and other salivary glands potentially can be further restricted. On the other hand, ART is considered to be an effective approach to correct anatomical variation and reduce the dose to OARs. Yet, it seems that not all patients are able to benefit from ART because it is challenged by time‐consuming procedures and requires extra resources. Additionally, the selection criteria for identifying patients who may benefit from ART are ambiguous, which obstructs the implementation of ART. The replanning strategies, timing, and optimal number of treatments remain unclear so far. Many researchers are investigating these issues, and some have made new progress.^15^, ^37^ We firmly believe that it will lead us to new important discoveries if these studies can be performed using CBCT.

## 5 CONCLUSION

Doses actually delivered to the parotid glands significantly increased compared with planning doses. Statistically significant differences in V_20_, V_30_, D_mean_, and D_50_ for parotid glands were found between patients with xerostomia and patients without xerostomia. Xerostomia is closely related to V_20_, V_30_, D_50_, and D_mean_. Elder patients were at high risk for developing xerostomia. Efforts to further restrict the radiation dose to the parotid glands are expected to have an effect on decreasing the incidence of xerostomia, especially for elder patients. Adaptive IMRT treatment planning using CBCT may be a useful way of treatment monitoring and optimization for head and neck cancer. The small number of patients limited our study. Independent and well‐designed studies including more patients are needed for further exploration.

## CONFLICTS OF INTEREST

The authors have no relevant conflicts of interest to disclose.
